# Globally aggregated biodiversity data impact predictive and descriptive research

**DOI:** 10.1073/pnas.2519119122

**Published:** 2025-12-09

**Authors:** Dirk Steinke, Birgit Gemeinholzer, Enrique Martínez-Meyer, Daniel Noesgaard, Andrew Young, Dmitry Schigel

**Affiliations:** ^a^Centre for Biodiversity Genomics, University of Guelph, Guelph, ON N1G 2W1, Canada; ^b^University Kassel, Faculty of Botany, Aufbau- und Verfügungszentrum (AVZ), Kassel 34132, Germany; ^c^Departamento de Zoología, Instituto de Biología, Universidad Nacional Autónoma de México, Ciudad de Mexico 14510, Mexico; ^d^Global Biodiversity Information Facility, Secretariat, København Ø 2100, Denmark; ^e^Centre of Australian National Biodiversity Research, Commonwealth Scientific and Industrial Research Organisation (CSIRO), Canberra, ACT 2601, Australia

**Keywords:** bibliographic analysis, occurrence records, GBIF, machine learning, data infrastructure

## Abstract

Here, we present an analysis of the growth and use of the Global Biodiversity Information Facility (GBIF) over the last 5 y. GBIF is the world’s largest data integrator for biodiversity information and plays a central role in research across the biodiversity and evolutionary science community. With the help of a comprehensive bibliographic dataset comprising 12,193 studies that used GBIF-mediated data, we demonstrate how the global scientific community utilizes the continuously fast-growing amount of open and Findable, Accessible, Interoperable, and Reusable biodiversity data in their research. Overall, more researchers engage with GBIF data, a potential consequence of the rising demands of more global environmental assessments, where GBIF-mediated data are being used as a key resource for biodiversity research. Studies utilizing species distribution modeling were most prevalent and data used for topics related to challenges of the Anthropocene (conservation, climate change, invasive, and pest species). More studies used observational data records, a category that also includes a substantial amount of citizen science data. Our data show that a thematic diversification of GBIF-using literature is accompanied by a rapid diversification of both the additional datasets that GBIF data are analyzed with, as well as the new analytical approaches taken by researchers. This emphasizes the growing importance of GBIF’s data infrastructure and services which support global sciences and reflect major shifts in applied science which dictate the need for GBIF and similar data infrastructures to evolve rapidly in order to maintain relevance for research.

## Significance

Given the increasingly central importance of data aggregators and infrastructures as critical resources for the global research community it is important to understand how they are evolving. Specifically, it would be useful to know how much they are growing, what new data streams they are ingesting, who is using the data they serve, how they analyze it and for what purpose? We used a comprehensive bibliographic dataset comprising some 12,000 studies that used Global Biodiversity Information Facility (GBIF)-mediated data to show how the global scientific community utilizes the continuously fast-growing amount of open biodiversity data in their research and underscore the collective responsibility to minimize data heterogeneity and advance data availability and data quality through gap filling and error correction.

As we reach the end of the first quarter of the 21st century, the role of science in providing accurate, relevant, and unbiased information from a variety of sources to develop evidence-based policy, and inform operational decision-making, has never been more important for the future of our species and our planet. This is true across a broad range of domains including food production, human health, energy distribution, biodiversity conservation, and ecosystem services. These increasingly complex tasks have created a need for massive, dynamic, curated, and connected data drawn from diverse information streams resulting in the rapid proliferation of domain-based global data integrators. These facilities source, integrate, and serve scientific data to the research community, generally using the FAIR (Findable, Accessible, Interoperable, and Reusable) principles of data management and sharing (1). Examples of major data infrastructure now include the Global Health Observatory (WHO) for health data, ONESTOP (NOAA) for environmental data, Opendata (World Bank) for global development data, FAOSTAT (FAO) for agricultural information, and the Global Biodiversity Information Facility (GBIF) for spatiotemporal evidence on life on Earth.

A quarter century ago, member states of the OECD officially launched GBIF as a distributed system of interlinked and interoperable modules (databases, software and networking tools, search engines, analytical algorithms, training etc.) to share biodiversity information of organisms and respective associated metadata. Cooperative structures were installed, supporting a global biodiversity data portal (GBIF.org) that consolidated various disconnected databases and mobilized data from institutions around the world. The portal was developed to better comprehend, protect, manage, and sustainably utilize the biological diversity of the world and to remove barriers that prevented cooperation in times of increasing threats to global biodiversity. Today, GBIF represents the world’s largest biodiversity information network, providing openly accessible, verifiable, and usable biodiversity data (1) from over 2,500 data publishers supported by a network of national and international participant nodes covering more than 120 countries.

Initially, GBIF focused on discovery and access to digital information connected to specimens stored at natural history collections (2) which was later fueled by extensive museum specimen digitization efforts (3–11). The next wave of data mobilization came through the integration of crowd sourced data especially from wide-spread observation-based platforms such as eBird (1.5 B occurrences by July 2025, 12), iNaturalist (~118 M occurrences, 13), and Observation.org (112 M occurrences, 14). In parallel, data generated for instance through the International Nucleotide Sequence Database Collaboration (INSDC) or the global DNA barcoding community (BOLD, 15 M occurrences, 15) provided primarily molecular data. In total, GBIF now provides access to more than 3 billion records and over 4 million accepted scientific names of living organisms.

More recently, GBIF began to see the significant influx of data resulting from recent advances in technology which allow for repeatable measurements of organismal occurrences at unprecedented scales. In particular, metagenomics (16), metabarcoding (17), environmental DNA (eDNA, 18), acoustic monitoring (19), camera trapping (20), and several other automated organismal detection systems (e.g., refs. 21 and 22) provide compelling advantages over traditional approaches for tracking shifts in species as each of them generate large volumes of georeferenced occurrence data at comparatively low cost.

GBIF has been leading efforts not only for data provisioning but also for data integration, e.g., soil and freshwater communities, vector organisms, and DNA derived data by producing guidelines for standardized incorporation of occurrence data (e.g., refs. 23–25) and piloting a program to simplify the process of publishing metabarcoding data to GBIF (www.gbif.org/metabarcoding).

Given the importance of major data infrastructure such as GBIF as critical resource for the global research community with growing demands for high data volumes, e.g., in macroecology, modeling of species distribution ranges, and deep learning algorithms, it is important to understand how such infrastructure is evolving. Specifically, it is useful to know how much these systems are growing, what new data streams are ingested, who is using the data that is served, how data are analyzed and for what purpose? Having answers to these questions will be very instructive in planning the future focus and growth of data aggregator activities if we are to maximize their value into the future as digital data engines of global research.

To address these questions, here we explore scientific literature using GBIF-mediated data to investigate the purpose of study and in which context data were analyzed. So far, only a few studies have investigated whether continued data aggregation within the GBIF network enabled novel, increasingly diverse, and global research (26), and how available biodiversity data have been utilized for fields of study not directly related to biodiversity research, e.g., human health (27). As GBIF data sources have expanded over time it begs the question whether and how this growth has impacted the type of studies the data supported and if there are trends in scientific fields and methods it applied.

In particular, machine learning approaches have been suggested as capable of transforming the identification and monitoring of species across the world, providing a novel tool in supporting action to understand and reverse biodiversity losses (e.g., ref. 28). However, it is unclear whether we really are amid a major transition in the field of biodiversity informatics. Machine learning algorithms, especially convolutional artificial neural networks (CANN) and large language models (LMMs), have emerged as effective methods for classification and object recognition (29–32). However, biodiversity datasets of sufficient size to properly train these models are scarce (32–34). GBIF is one of the few data infrastructures that serve the adequate amount of data to fulfil such requirements, however, data are spatially and taxonomically skewed and the question remaining is if users are utilizing GBIF-mediated data accordingly and how data licensing and data citation will develop in the context of training the models.

This study is the second (26) comprehensive analysis of literature-based data use patterns of GBIF and goes beyond the scope of former work by also I) documenting changes in usage patterns over time to determine general trends, II) exploring analysis methods used and linking them to data sizes and types, III) understanding how the scientific community responds to the growing availability of GBIF-mediated data especially to a wider variety of data sources. Understanding these trends sheds light on continued and emerging applications of GBIF information as well as the data environment in which they are analyzed. The analyses provide information to support the development of both GBIF data content and infrastructure. They outline the kinds of information GBIF should incorporate and the tools GBIF provides while also offering insights into the added value of global data aggregation and provision.

## Results

### General Patterns and Geographic Breakdown.

**Fig. 1.**
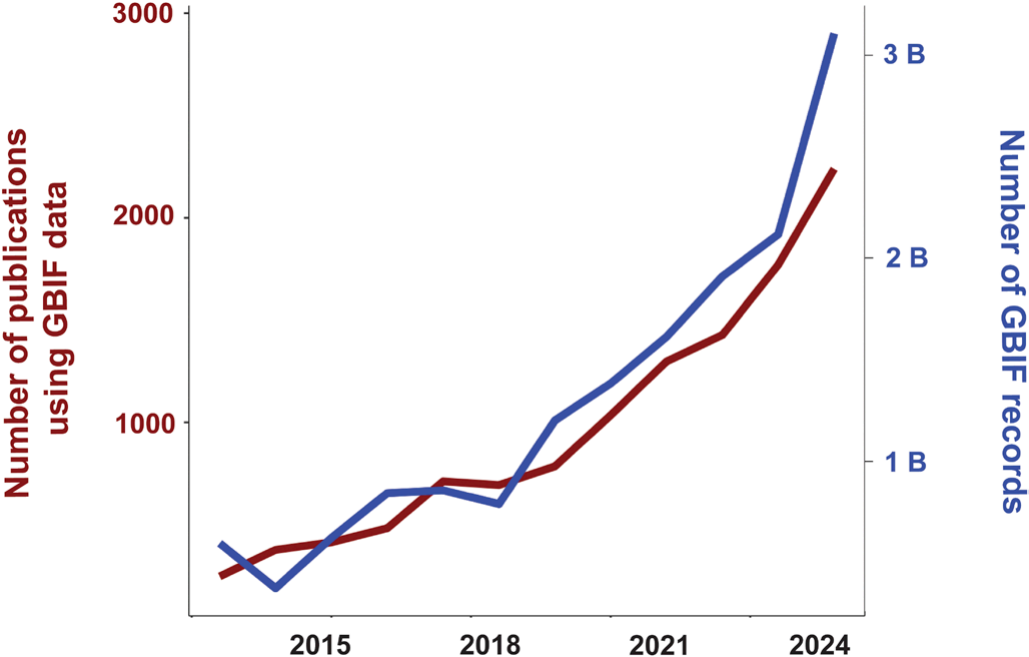
Growth over time of the biodiversity occurrence data accessible via the GBIF and peer-reviewed articles using these data.

**Fig. 2.**
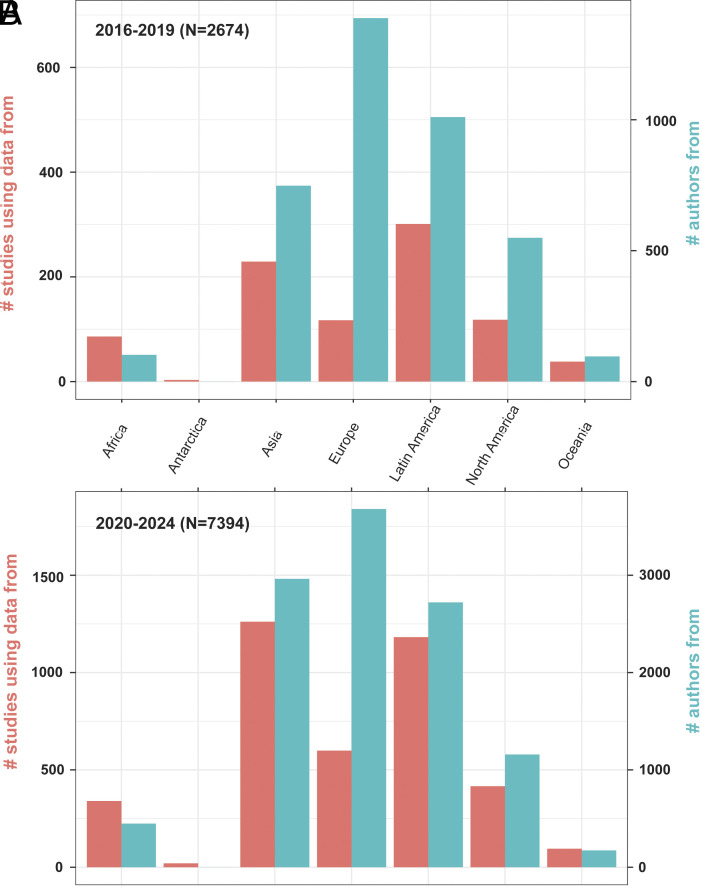
Number of studies by geographic origin, and number of authors by country of affiliation for earlier (*A* – 2016-2019) and more recently published studies (*B* – 2020-2024).

A total of 12,193 peer-reviewed journal articles using GBIF-mediated data were extracted from GBIF’s literature tracking database. Of these, 7,786 were published in the years 2020–2024 representing a significant increase in the rate of use following past trajectories of database growth: the count of GBIF using peer-reviewed publications more than doubled since 2019 while the number of GBIF accessible records almost tripled—Fig. 1). GBIF data-mediated publications from 2016 to 2024 utilized records from 169 countries. The top five data producers were China, United States, Mexico, Brazil, and Colombia (*SI Appendix,* Table S1). Occurrence records from 158 countries were cited for the 2020–2024 period, 101 for 2016–2019 representing a geographic expansion of 36% over the past 5 y. The pattern of geographic coverage of publications among GBIF regions did not change considerably over the past 5 y, as all appear to grow at proportional rates (Fig. 2).

Authors of studies within the 2020–2024 period were affiliated with institutions in 142 countries, mostly in the United States, China, United Kingdom, Brazil, and Mexico (*SI Appendix,* Table S2). The data show the largest increase of affiliations over the past 5 y for the Europe (N = 3,680), and the Asia (N = 2,963), followed by Latin America (N = 2,721) and North America (N = 1,158) (Fig. 2).

### Main Fields of Study.

**Fig. 3.**
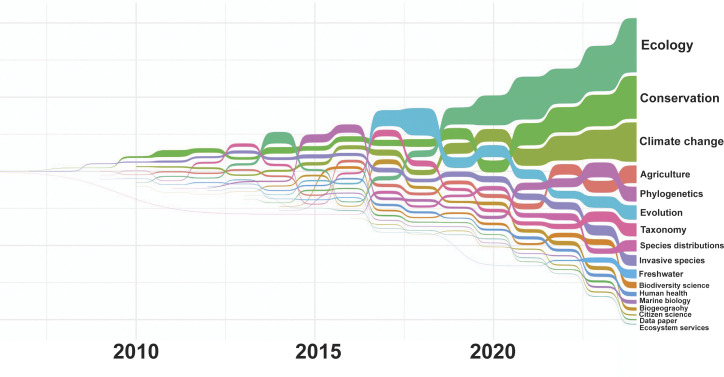
Sankey bump plot depicting the change of main publication research topics (*SI Appendix,* Table S4) over time.

Studies were mostly focused on biodiversity in combination with climate change, invasive species, conservation, evolution, ecology, and biogeography. Prevalences have changed over time (Fig. 3 and *SI Appendix,* Fig. S2), especially biodiversity in combination with climate change (since 2019) and invasive species (jump in 2023) grew over time. Species distributions used to be the dominant topic until 2018 but now moved to rank 6.

**Fig. 4.**
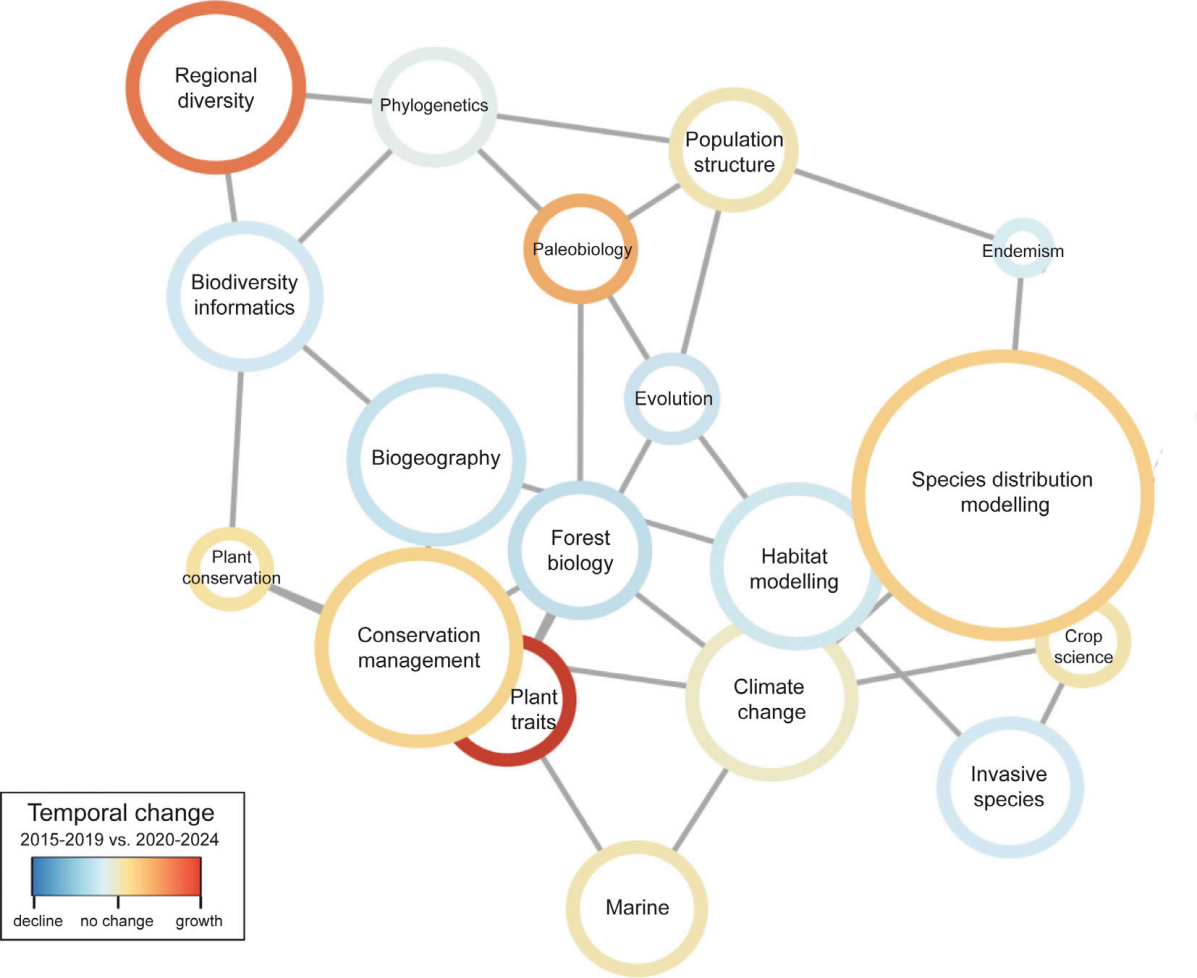
Structural topic model results from 4,301 studies that used GBIF-mediated data published from 2020 to 2024. Topic correlations network visualizes quantitative associations between topics (nodes), with topics near each other and connected by a gray line more likely to appear together in a given study. Node color denotes the relative change in prevalence over time within each topic, comparing topic prevalence in earlier studies (2016 to 2019) to those recently published (2020 to 2024). Node sizes are proportional to overall topic proportions.

We used correlation network analysis to visualize biodiversity related research topic clusters (Fig. 4). Related topics are those that comprise word sets extracted from title, keywords, abstract, and main text that are shared within and across studies. Accounting for 38% of the literature, the top 6 most prevalent biodiversity-related topics related to aspects of geographic distribution of species, climate change, and conservation management. Topics relating to conservation management clustered together with topics relating to plants and forests. Another cluster included modeling, climate change, and research on harmful species (invasives and pests). We compared relative differences in overall topic prevalence through time by comparing studies published from 2016 to 2019 summarized in the preceding publication (26), to those published between 2020 and 2024 (Fig. 4 and *SI Appendix,* Figs. S3 and S4 and Table S3).

Articles from 2016 to 2024 using GBIF-mediated data were published in 1,895 peer-review journals, of which 33% were open access at time of publication of the respective study. A comparison between the time periods of 2016–2019 and 2020–2024 showed an ongoing trend toward large multidisciplinary journals (e.g., Ecology & Evolution, Scientific Reports, PLoS ONE (*SI Appendix,* Fig. S5). Other members of the top 50 journals continued to be mostly conservation and ecology themed.

### Data Sources.

**Fig. 5.**
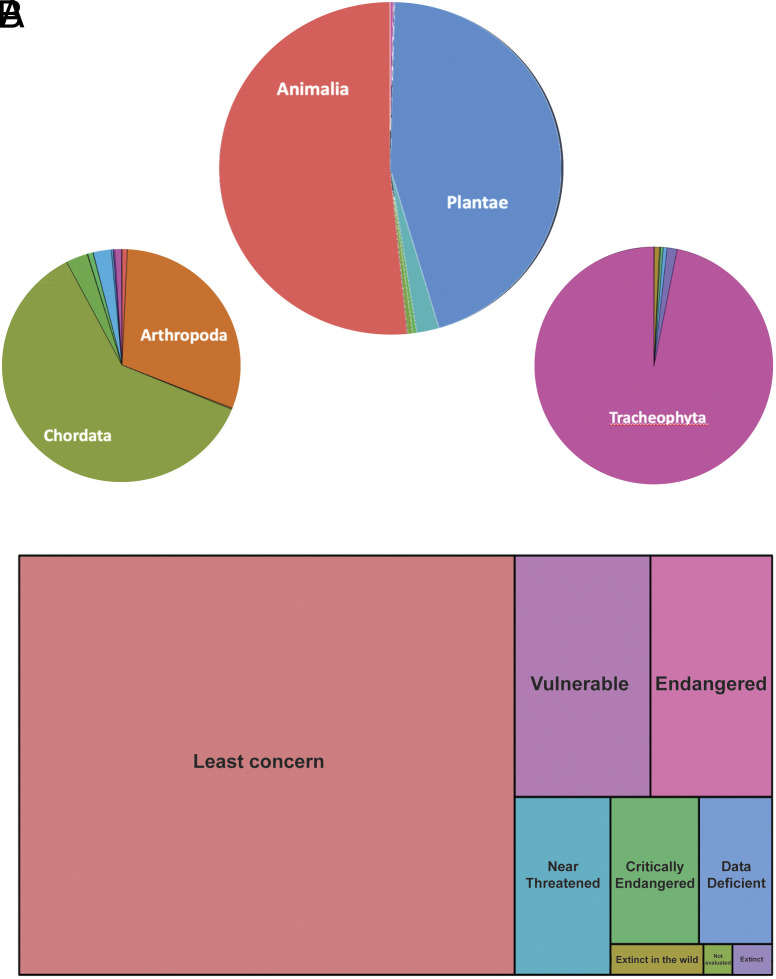
Taxonomic distribution across studies using GBIF-mediated data. (*A*) Shows pie charts for number of publications by kingdom (*Top*), animal phyla (*Bottom Left*), and plant phyla (*Bottom Right*). (*B*) Depicts a treemap of species count per IUCN risk category.

Most studies focused on animals (80%) and plants (19%) (Fig. 5*A*). Most animal studies focused on chordates and arthropods (Fig. 5*B*). Almost all studies on plants involved tracheophytes (Fig. 5*C*). Within the investigated pool from 2016 to 2024, we found over 6,000 studies that included species listed on the IUCN Red List (N = 6,748, Fig. 5*D*), most of them falling under “least concern” but a considerable number of species fell within one of the threat categories (N = 1,996) and a few studies included extinct taxa (N = 68).

**Fig. 6.**
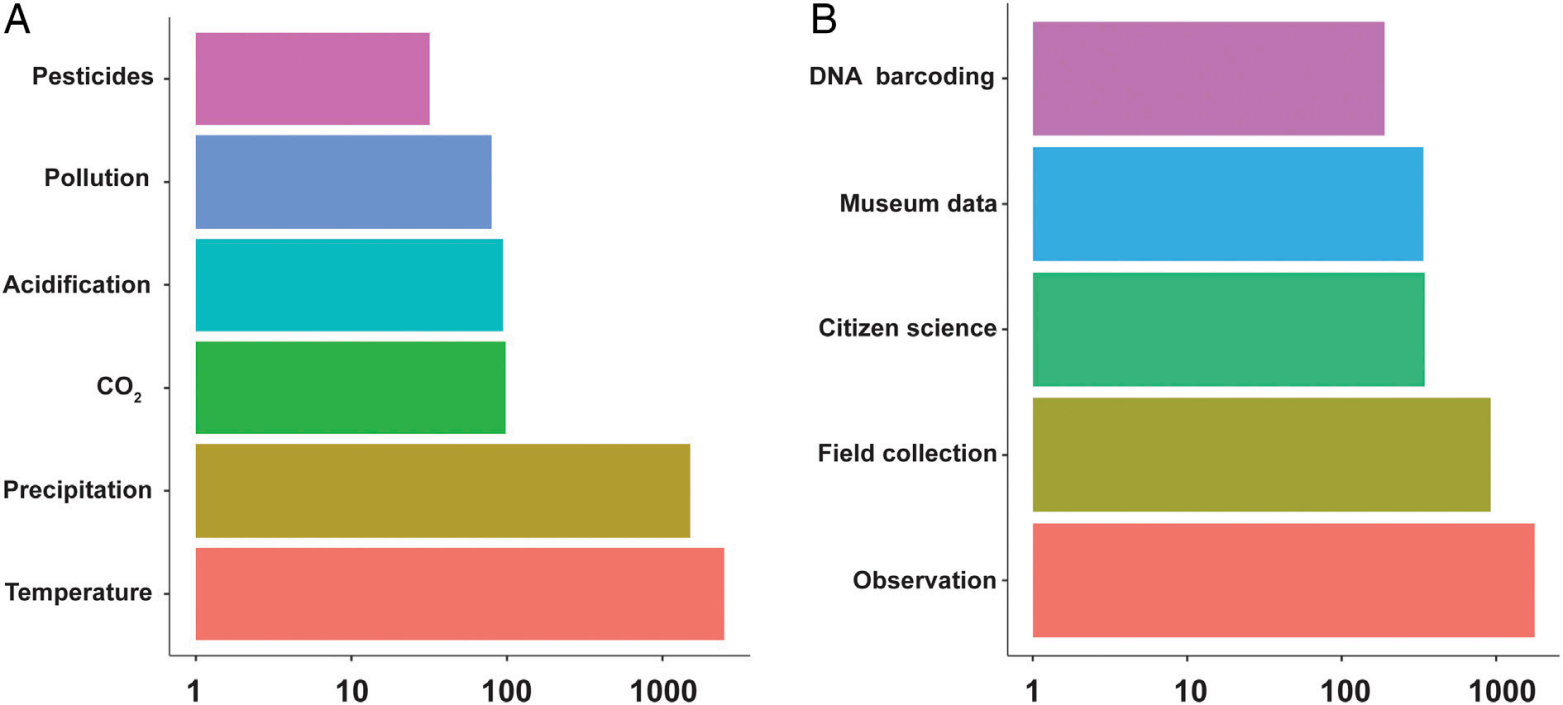
Logarithmic barplots showing (*A*) other data categories analyzed with GBIF-mediated data. and (*B*) sources of occurrence records.

GBIF-mediated occurrence records are often analyzed alongside other types of (abiotic) data. Most studies found in this context used temperature (N = 2,478) and precipitation (N = 1,506), followed by CO_2_ concentration and measures of acidification (pH) (N = 98). Various pollution measures (N = 80) and pesticides (N = 32) were used less often (Fig. 6*A*). Most publications used observational data and field collection results (expeditions/surveys). Museum collections were also used as well as citizen science data (largely iNaturalist). Publications using DNA barcoding data (N = 189) included papers that used metabarcoding (35 studies) and eDNA data (27 studies) (Fig. 6*B*). GBIF provides access to about 15 M iBOL occurrence records which is about 98% of BOLDs public database. In contrast, only 500 K occurrence records extracted from DNA metabarcoding studies (eDNA and community samples) can be found on GBIF. Given that there are about 8,000 metabarcoding publications (Google Scholar, April 2025) it is conceivable that at least 100 M occurrence records could be extracted from published data sources of these publications.

The GBIF API provides a programmatic way to query GBIF-mediated data. As APIs are increasingly used to gain access to larger data quantities, we wanted to explore whether studies use them more often. One way of indirectly measuring the impact of the API is by tracking publications/studies using packages that help users to utilize it. For R, this would be rgbif, which had a total of 449 direct citations (Google Scholar, April 2025) since its publication in 2022 (plus 146 for the paper). There is also a Python version (pyGBIF) which showed 179 citations.

### Methods Used in Studies.

**Fig. 7.**
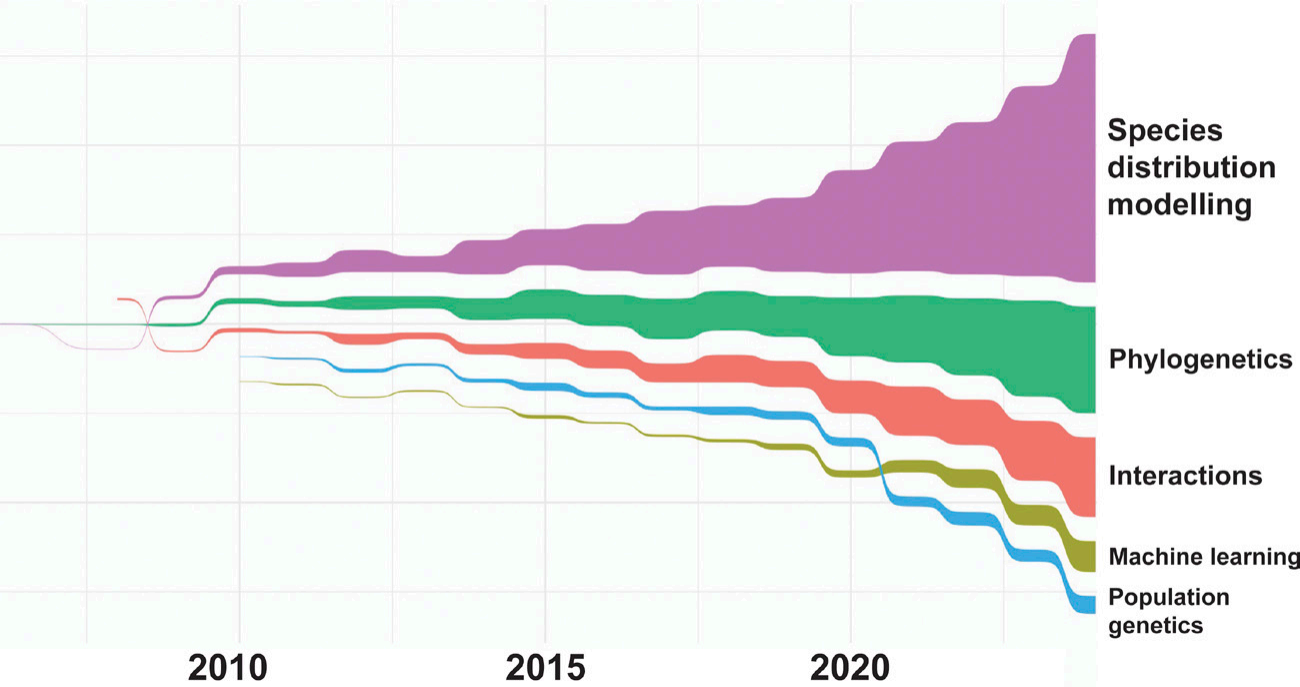
Sankey bump plot depicting the change of methods used in studies utilizing GBIF-mediated data.

We selected the five most dominant groups of methods used in publications using GBIF-mediated data (for N = 5,400 studies). Fig. 7 shows the numbers of publications for each of those groups over time. Species distribution modeling is dominating the list (2,604 publications), followed by phylogenetics (1,418) and interaction analyses (882). The latter comprises nontrophic and trophic interactions. All three groups show constant growth over the past decades, but in the last 2 y, the latter two groups remained unchanged. Notably, machine learning approaches have been on the rise over the past few years (239). At this point, the number of publications represents only 2% of the overall total, but it is conceivable that it will increase rapidly. About 36% of machine learning use is related to species distribution modeling, 13% to phylogenetics, and 6.2% to interaction analysis. Population genetics studies are using GBIF-mediated data at a constant rate (242 total), but no growth could be observed.

## Discussion

As the complexity of the challenges we face continues to grow, the responses and actions required to meet them at scale, and with sufficient timeliness, require access to more data, from a broader range of sources. These need to be harmonized so they can be interrogated jointly using the next generation of data analytics and more sophisticated models, including AI. The joint lasting efforts for reliable data aggregators to integrate trustworthy information support GBIF, the world’s largest biodiversity data provider (1), providing a case to assess the impact of large-scaled mobilization efforts on current research. With the help of a comprehensive bibliographic dataset comprising 12,193 studies that used GBIF-mediated data, we were particularly interested in how the global scientific community utilizes the continuously fast-growing amount of open and FAIR biodiversity data in their research. The body of available literature has more than doubled over the past 5 y, while the number of records available on GBIF has tripled. It is noteworthy that the preceding study (26) analyzed a third of the current study count (N = 4,035). This growth of published knowledge over just 5 y as well as the expansion of GBIF-mediated data (Fig. 1) prove the importance of the data infrastructure and its acceptance within the research community.

Overall, more researchers engage with GBIF-mediated data, a consequence of the fast growth of available GBIF records and potentially the rising demands of more global environmental assessments, where GBIF-mediated data are being used as a key resource for biodiversity research (35, 36). For example, the IPBES report (36) included 130 references that used GBIF-mediated data. These papers provided scientific support for 31 of the 32 background messages of the report.

Authorship seems to be moving away from a North American-centric to a European-centric distribution of data use. Although authorship especially in Asia and Latin America increased, there remains a contrast when it comes to data origin especially for Latin America and Africa (Fig. 2). Research on the diversity of these regions is still authored more by researchers especially from Europe, a phenomenon that is well known (e.g., refs. 37 and 38).

### Main Fields of Study.

Earlier bibliometric studies found that the most common uses of online biodiversity databases have been to estimate species distribution and richness (26, 39). Despite still being among the top six topics, species distributions became less relevant in comparison with other topics such as ecology, conservation, or climate change. Not surprisingly, our analyses showed increased data use for hot topics of the age of the Anthropocene (conservation, climate change, invasive, and pest species). The prevalence of these topics suggests that studies are increasingly driven by societal relevance and potential application, but interestingly, explicit human health uses showed no further growth contradicting a trend described in ref. 27 which was largely driven by the COVID pandemic. Another cluster comprises fields related to conservation biology, again emphasizing the more applied usage of GBIF-mediated data. (both descriptive and predictive, less basic science, more applied science, e.g., ecological monitoring and nature conservation). It is possible that our data are overemphasizing certain trends and underestimate others because GBIF topics are technical categories used for literature tracking purposes which means they are not fully congruent with commonly used scientific discipline definitions.

### Data Sources.

Use of GBIF-mediated data is driven by availability of taxa and the application of heterogenous data. While we observe general growth of data quality, there is no doubt that globally aggregated biodiversity data will remain heterogeneous. GBIF is rolling out the next interface (preview available at https://demo.gbif.org) which takes the principal approach of building more efficient flagging and filtering instruments across its data access products and of building capacity in data cleaning and data fitness for use.

Overall, GBIF largely consists of chordate and of tracheophyte records, and this is reflected in the taxonomic focus of studies that continue to draw on GBIF as primary data source. However, independent from the taxonomic source, particular attention is paid to species of conservation concern, for which data are obscured to ensure that endangered species are not compromised. For instance, almost half of the reviewed studies published 2016–2024 included data on species listed on the IUCN Red List, which is also skewed taxonomically, with almost one third of those falling into one of the threat categories. This corroborates the trend of GBIF-data being more heavily used in conservation research, management, and application.

Most studies utilizing GBIF-mediated occurrence records used observational data, a category that also includes a substantial amount of citizen science data (e.g., iNaturalist). The growing public interest in sharing biodiversity observations has led to a significant increase in initiatives that connect citizen scientists and researchers. This is an interesting development as until recently citizen science–sourced biodiversity data featured only in a relatively small percentage of research papers on biodiversity (40).

As biodiversity studies often use a combination of data types, including both qualitative and quantitative data an interesting question is what other information types GBIF-mediated data studies were using. Climate variables such as temperature and precipitation were the most included and integrated into analyses, as well as more directly measured human-induced factors such as CO_2_, acidification, pollution, and pesticides. The former are traditionally part of distribution and habitat modeling, while the latter have been only recently being brought into this field, especially in the context of conservation management.

### Methods Used in Studies.

Species distribution modeling continues to be the most frequently used method in GBIF-mediated data studies even though the application is not solely focussing on species distributions per se but on more applied problems in the context of conservation and predictive frameworks (41). GBIF data also continue to represent important input data for descriptive studies utilizing phylogenetic methods such as diversity estimates that consider the evolutionary relationships among species (42) as well as interaction analyses that, for instance, calculate dietary niche width and trophic position of organisms in large food networks (43).

Methods using AI promise to provide innovative solutions for many challenges. This does include the field of biodiversity science where, for instance, the analysis of long time series and spatial dynamics as well as comprehensive network analyses have been identified as future areas of application (44). Especially, the predictive power of AI methods can help to model possible future scenarios and to assess conservation measures (45), however, it needs to be noted that not only available but also missing data influence results. Over time, GBIF has accumulated suitable data that could be used for such studies, but the challenge will be to serve them in formats conducive to machine learning approaches, and in the way, that data-generating and data-interpreting communities accept as ethical and fair. Despite increased use of machine learning in the field of biodiversity science, our results suggest that by the end of 2024, it has not yet arrived in studies that utilize GBIF-mediated data. The lack of detection of a large increase in applications of machine learning during 2020–2024 might be partially masked in studies utilizing SDMs, which are increasingly done using Random Forest models (e.g., ref. 41). However, this might also have to do with the fact that most current AI applications in biodiversity science are using other data types, such as images (e.g., ref. 46) and ecoacoustic monitoring files (47) which are not aggregated through GBIF but only supplement the dependent biodiversity data.

### Outlook.

The importance of data-driven decisions and actions has become increasingly clear over the past decade. Nations are turning to science to provide real-time solutions to complex, large-scale problems such as global pandemics, food shortages, and loss of biodiversity. Simultaneously, the rapid expansion of digital connectivity has improved our ability to source, to integrate, and to analyze scientific data at scale to chart the way forward in this ever more complex decision-making environment. The existence and development of global data aggregators have become a key part of the research community’s response to these challenges, but as illustrated here, it is important to understand more deeply how they are being adopted and used if they are to fully realize their potential. In this context, the availability of past data enables critical validation analyses for verification and for studies of biodiversity change.

GBIF has grown and evolved in terms of the volume and taxonomic breadth of data that it aggregates and the variety of data sources it relies on which has expanded from museum specimens and citizen science observations to include eDNA and other emerging data streams such as ecoacoustics and camera trap data. At the same time, the application space for GBIF data has also changed and broadened to include OneHealth (27), tourism (48), urban development (49), agriculture (50), and increasingly climate change (51). Thematic diversification of GBIF-using literature has been accompanied by a rapid diversification of both the additional datasets that GBIF data are analyzed with, as well as the new analytical approaches taken by researchers (52). These trends underscore the growing importance of GBIF’s data infrastructure and services which support global sciences and reflect major shifts in applied science toward areas such as climate change response, biosecurity, disease preparedness, and food security. The GBIF network inspires other distributed network structures, on national scales with many emerging national biodiversity data centers (53) but also beyond for other disciplines, e.g., infectious diseases (ISIDORE).

In this context, particular challenges for GBIF are likely to include 1) streamlining data input workflows to support the integration of emerging data streams, in particular scalable remote data sources such as ecoacoustics and camera-based biodiversity observations; 2) ensuring scalability as new data layers become available for existing data points, e.g., addition of digital sequence information to specimen-based data, or additional environmental information for eDNA samples and; 3) continuing capacity building, including data cleaning and data fitness for use; 4) enhancing interoperability with other large global datasets that represent potential drivers of biodiversity into the future including climate, land use, and socioeconomic data.

In short, the dynamic nature of data-driven research dictates the need for data infrastructures to evolve rapidly in order to maintain relevance for research.

## Materials and Methods

### Information Extraction.

For this study, we utilized both the GBIF bibliographic database and GBIF’s Global data trends as main resources (*SI Appendix,* Fig. S1). Since 2010, the GBIF Secretariat has tracked use of GBIF-mediated data in all forms of literature, including scholarly dissertations, government reports, and others, with a primary focus on peer-reviewed journal articles. The GBIF literature tracking programme has evolved from a largely manual effort, capturing no more than a few papers every week on average in the first years to an automatic process that captures, curates, and stores bibliographic data for at least one paper per day. In 2024, the literature programme captured on average more than six peer-reviewed articles using GBIF-mediated data per day.

In brief, GBIF-relevant keyword alerts from various scholarly databases (e.g., Google Scholar) and journal publishers form the basis of the literature-tracking programme. As such, every single paper published mentioning GBIF will ideally feed into the literature pipeline. Most alerts are email-based and funnelled through a parser to extract relevant details automatically upon receipt. Regardless of source, the potential hits are all stored in a temporary database for manual assessment. GBIF Secretariat staff processes entries in the queue daily by eliminating false positives and curating bona fide use cases. The curation process involves extracting article metadata while decorating entries with additional information, such as topics based on GBIF-assigned science categories (*SI Appendix,* Table S4), geography covered, and links to datasets and taxa. Once fully curated, true positives are added to the bibliographic database, which is accessible via web (https://www.gbif.org/resource/search?contentType=literature) and REST API (https://techdocs.gbif.org/en/openapi/v1/literature). For this study, we used the literature REST API to extract all peer-reviewed journal articles using GBIF-mediated data by applying the filters literature Type (=journal), relevance (=GBIF_USED), and peerReview (=true). (retrieved on Feb 12, 2025).

### Data Analysis.

We utilized the extracted metadata to generate figures that show geographic distribution of authorship and data origin over time (*ggplot2,* 54) in R (55). In order to extract deeper context from the publications, we used the SciSpace Extract Data function (https://typeset.io/extract-data), which uses LLMs to extract more complex information from scientific publications. For this analysis, we downloaded full text pdf files in accessible format (N = 4,301) using DOI links provided through the GBIF bibliographic database (*SI Appendix,* Fig. S1). Subsequent automated text analysis was performed on 10,482 articles, including Scispace results (summary, conclusions) where available, article abstracts, titles, and keywords using the *stm* package (56) in R (55). Publication year was included as a covariate. We modified scripts from ref. 26 to incorporate Scispace results into the *stm* analyses and, at the same time, extract information on taxonomy, data sources (both GBIF occurrence records and associated variables analyzed), and methodology using keyword searches. In case of multiple category hits, all were counted. The latter set was either used to generate figures showing their usage over time or to build bar charts using *ggplot2* and Sankey charts using *ggsankey* (https://github.com/pepijn-devries/ggsankeyfier/).

To better understand the choice of study organisms in the context of conservation studies, we used extracted species-level taxonomy to assign IUCN Red List categories to bibliographic records for which appropriate taxonomy was available (N = 4,429). For this, we utilized the checklist dataset available through GBIF (https://doi.org/10.15468/0qnb58) (57). Treemaps depicting Red List category distribution were built using *treemap* (58). All R scripts used are available through Zenodo (10.5281/zenodo.15994577).

## Supplementary Material

Appendix 01 (PDF)

## Data Availability

Previously published data were used for this work (https://doi.org/10.15468/0qnb58) (57). All other data are included in the manuscript and/or *SI Appendix*.
