# Supplementary material for: Globally aggregated biodiversity data impact predictive and descriptive research
Source: Proc Natl Acad Sci U S A. 2025 Dec 9;122(50):e2519119122. doi: 10.1073/pnas.2519119122 (PMC12718365; doi:10.1073/pnas.2519119122)
Supplement: Supplementary file 1 — Appendix 01 (PDF) [file pnas.2519119122.sapp.pdf]

Supplementary data

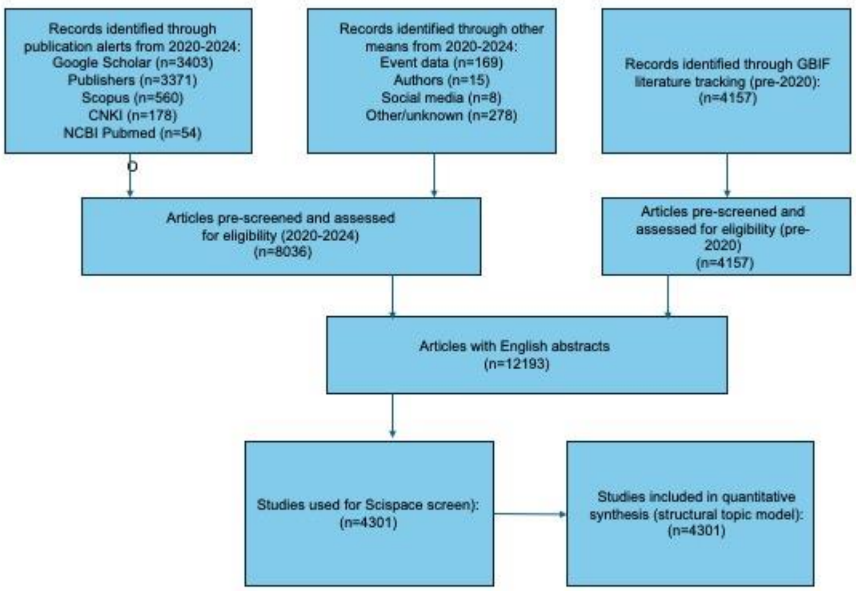

Supplementary Figure 1: PRISMA flow chart outlining process of literature compilation for inclusion in bibliometric analysis and topic models.

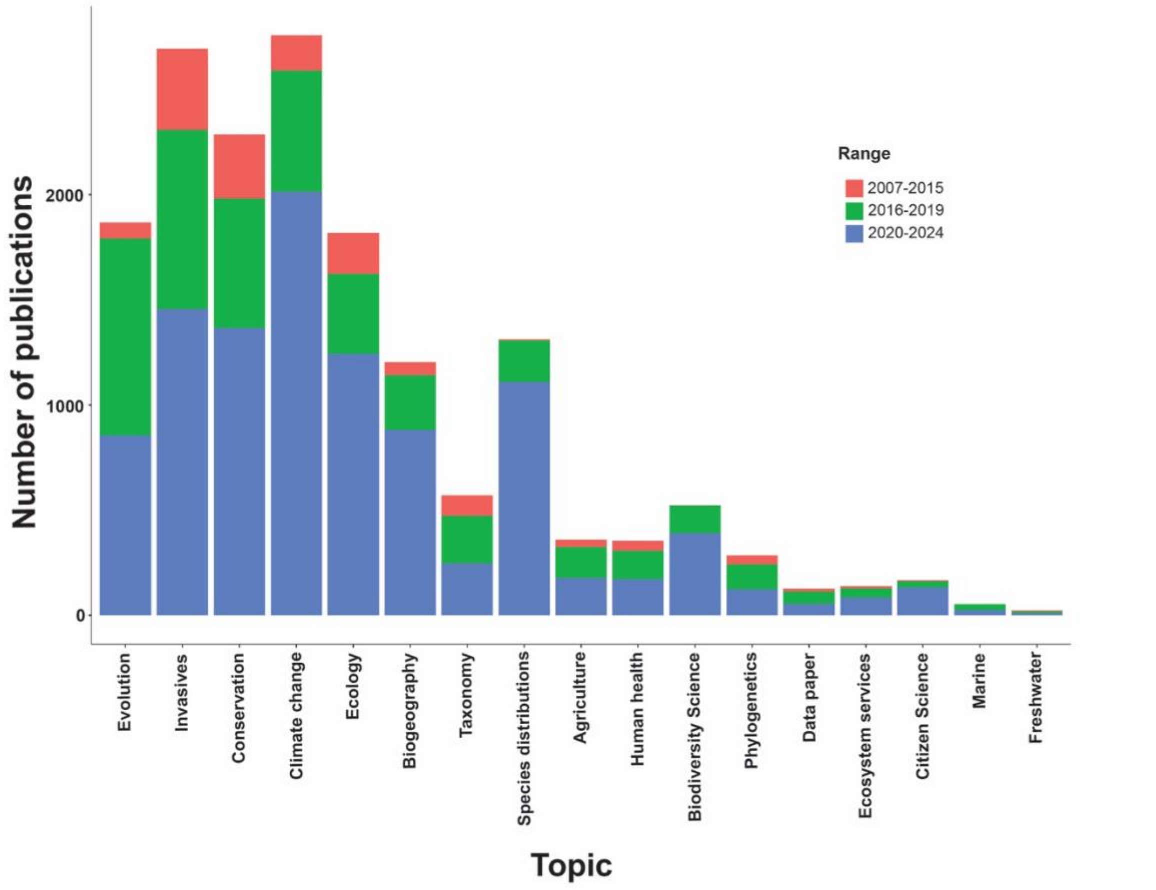

Supplementary Figure 2: Stacked bar chart showing number of publications by general topic (ranges: 2007-2015, 2016-2019, 2020-2024)

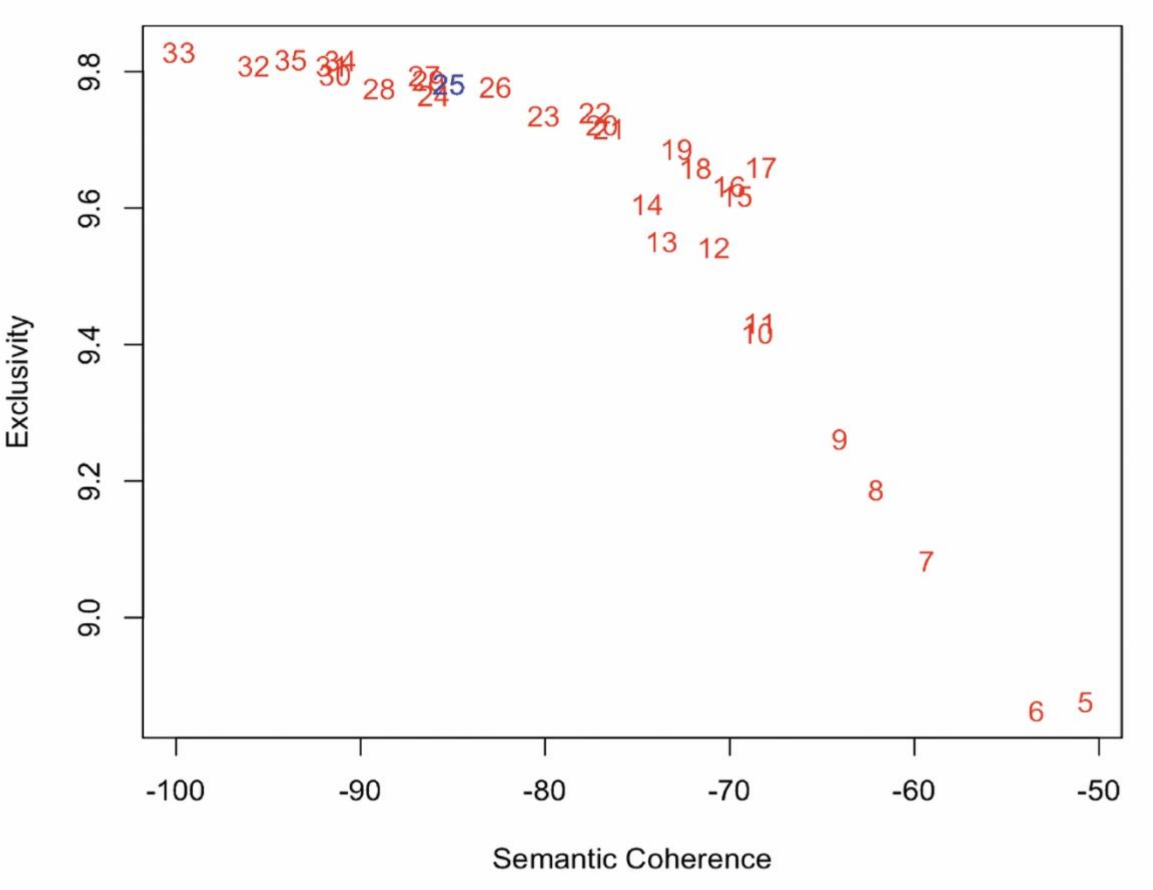

Supplementary Figure 3: Relationship between model exclusivity and semantic coherence. Each number refers to a separate topic model that included that number of topics (K). The number's location on the graph corresponds to the average values for that topic model for exclusivity and semantic coherence. Because topic modeling estimation is not deterministic, models were run twice for each number of topics to ensure conclusions were consistent across model runs (model runs denoted by different colors – in case of full congruence, red is the visible colour).

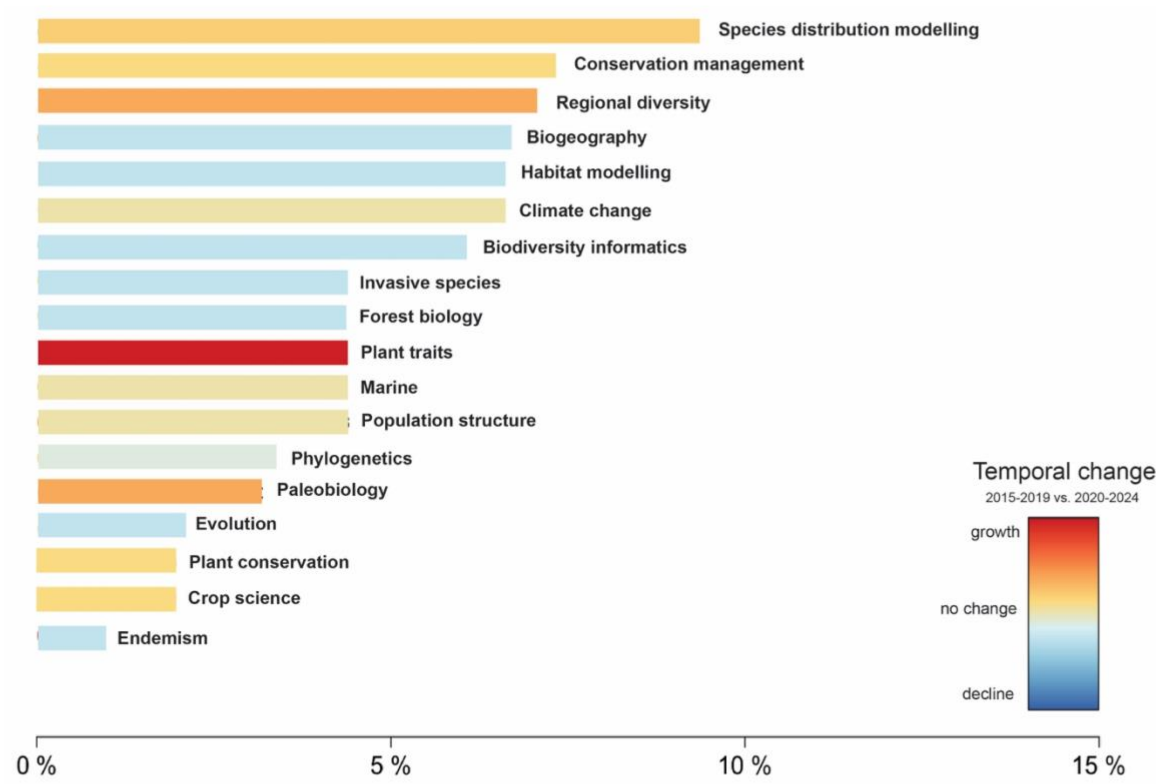

Supplementary Figure 4: Topic proportions from 18-topic structural topic model of GBIF-meditated studies. Topic proportions are the percentage of the total corpus classified to each topic.

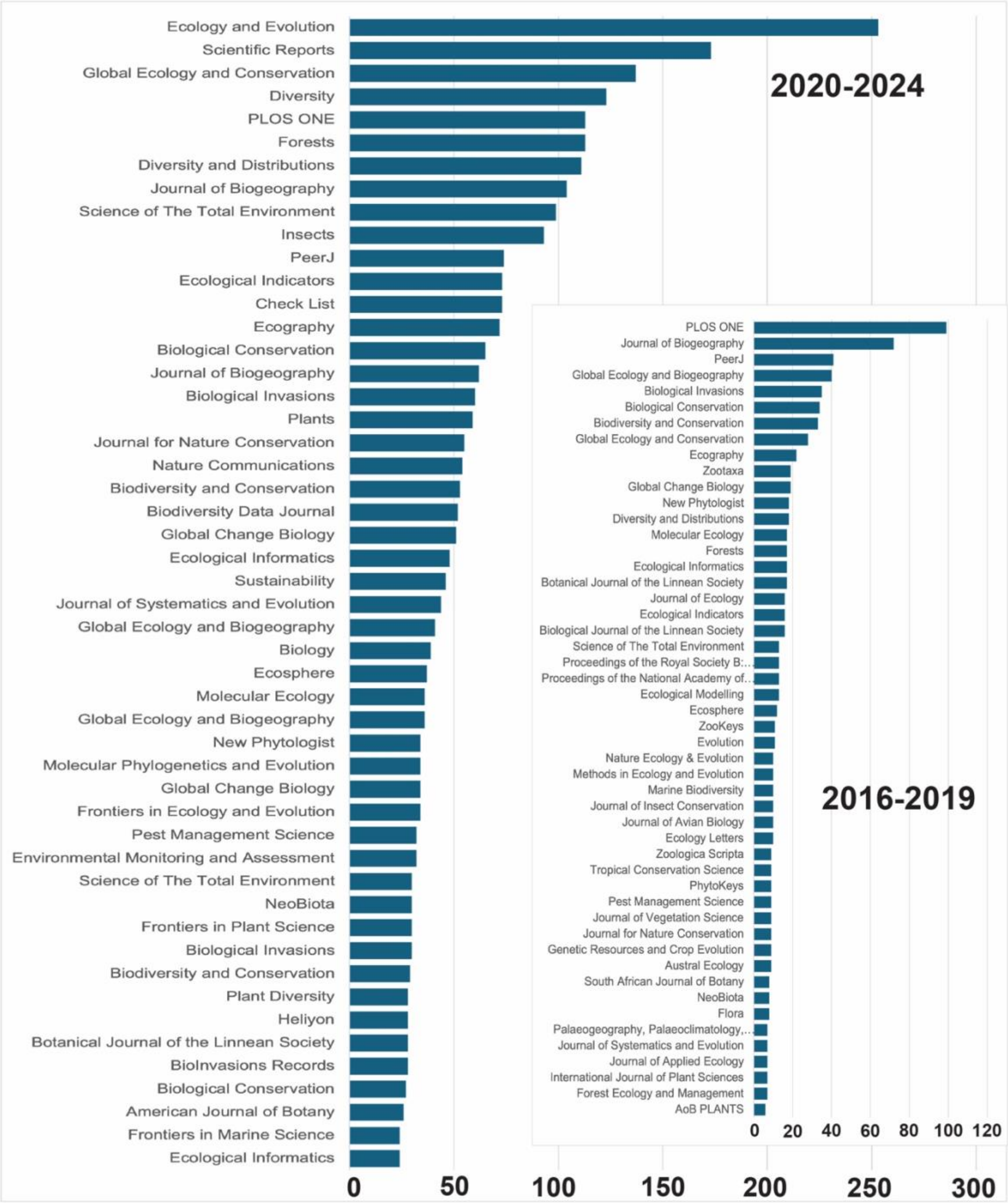

Supplementary Figure 5: Number of GBIF-mediated articles published in the 50 most common journals published from 2016 to 2019 and from 2020-2024.

Supplementary Table 1: Origin of data in GBIF mediated publications (including data available through GBIF)

Country of Origin	#publications
China	856
United States	615
Mexico	461
Brazil	441
Colombia	151
India	144
Argentina	114
Australia	106
Portugal	104
South Africa	100
Spain	87
Canada	66
Indonesia	58
New Zealand	58
Russia	58
Italy	57
United Kingdom	55
Iran	53
Chile	49
Ecuador	49
South Korea	48
Peru	44
Sweden	43
Norway	42
Japan	38
Turkey	37
Ethiopia	34
Benin	32
Antarctica	31
Madagascar	29
Kenya	28
Costa Rica	27
Taiwan	26
Uruguay	26
France	24
Sri Lanka	23

Philippines	23
Nepal	22
Pakistan	22
Ukraine	22
Laos	21
Algeria	20
Malaysia	19
Germany	18
Greece	18
Bangladesh	16
Belgium	16
Papua New Guinea	16
Romania	15
Poland	14
Thailand	14
Austria	13
Switzerland	13
Morocco	13
Panama	13
Burkina Faso	12
Cuba	12
Ireland	12
Mozambique	12
Namibia	16
El Salvador	12
Uganda	12
Egypt	11
Nigeria	11
Tanzania	10
Vietnam	10
Israel	9
Serbia	9
Singapore	9
Tajikistan	9
Angola	8
Brunei	8
Central African Republic	8
Gabon	8
Iraq	8

North Korea	8
Mongolia	8
Puerto Rico	8
Armenia	7
Dominican Republic	7
Honduras	7
Iceland	7
Myanmar (Burma)	7
New Caledonia	7
Sierra Leone	7
Uzbekistan	7
Venezuela	7
Bolivia	6
Estonia	6
Finland	6
Greenland	6
Guatemala	6
Hong Kong SAR China	6
Latvia	6
São Tomé & Príncipe	6
Georgia	5
Ghana	5
Croatia	5
Kazakhstan	5
Lebanon	5
Libya	5
Niger	5
Nicaragua	5
Netherlands	5
Saudi Arabia	5
Togo	5
Tunisia	5
Bosnia & Herzegovina	4
Bulgaria	4
Cameroon	4
Denmark	4
French Guiana	4
Montenegro	4
Oman	4

Paraguay	4
Samoa	4
Bhutan	3
Botswana	3
Congo - Kinshasa	3
Cote d'Ivoire	3
Cape Verde	3
Fiji	3
Guinea	3
Malawi	3
French Polynesia	3
Slovenia	3
Zimbabwe	3
Andorra	2
Cyprus	2
Czechia	2
Eritrea	2
Guyana	2
North Macedonia	2
Mauritania	2
Malta	2
Palestinian Territories	2
Sudan	2
Slovakia	2
Senegal	2
Syria	2
Chad	2
United States Minor Outlying Islands	2
Afghanistan	1
Albania	1
Azerbaijan	1
Belarus	1
Belize	1
Djibouti	1
Micronesia (Federated States of)	1
Equatorial Guinea	1
Haiti	1
Hungary	1
Jamaica	1

Jordan	1
Kyrgyzstan	1
Lithuania	1
Luxembourg	1
Mali	1
Qatar	1
Reunion	1
Rwanda	1
Solomon Islands	1
Svalbard & Jan Mayen	1
Suriname	1
South Sudan	1
Eswatini	1
French Southern Territories	1
U.S. Virgin Islands	1
Zambia	1

Supplementary Table 2: Country of authors of GBIF-mediated publications

Country of Authors	#publications
United States	3736
China	2763
United Kingdom	904
Brazil	888
Mexico	723
Portugal	644
Australia	617
Sweden	485
Argentina	348
South Africa	346
Germany	320
Italy	318
New Zealand	275
India	270
Spain	243
Switzerland	202
Colombia	188
Kenya	184
Austria	148
France	148
Canada	144
Russia	134
Japan	133
Czechia	131
Chile	129
Belgium	127
Norway	126
South Korea	108
Iran	90
Finland	87
Poland	85
Turkey	78
Denmark	69
Indonesia	50
Saudi Arabia	50
Ecuador	47

Serbia	47
Taiwan	43
Benin	40
Ethiopia	40
Estonia	37
Sri Lanka	37
Uruguay	36
Latvia	32
Kuwait	30
Netherlands	29
Ukraine	25
Burkina Faso	24
Ireland	24
Namibia	24
Brunei	23
Costa Rica	23
Philippines	23
Slovakia	23
Algeria	22
Hungary	22
Israel	22
Morocco	22
Singapore	22
Peru	21
Tunisia	21
Cuba	19
Egypt	18
Luxembourg	18
Greece	17
Pakistan	17
Romania	17
Bosnia & Herzegovina	16
Iraq	14
Laos	14
Libya	14
Venezuela	14
Malaysia	12
Niger	12
Thailand	12

Uzbekistan	11
Bangladesh	10
Malta	10
New Caledonia	10
Nicaragua	10
El Salvador	10
Lithuania	9
Nigeria	9
Nepal	9
Armenia	8
Bulgaria	8
Gabon	8
Iceland	8
Kazakhstan	8
Panama	8
Qatar	8
Bolivia	7
Slovenia	7
Zimbabwe	7
Dominican Republic	6
Hong Kong SAR China	6
Honduras	6
Mongolia	6
Malawi	6
Belarus	5
Cameroon	5
Croatia	5
Uganda	5
Cote d'Ivoire	4
Jordan	4
Cambodia	4
Lebanon	4
North Macedonia	4
Togo	4
Tanzania	4
Vietnam	4
Ghana	3
Guatemala	3
Montenegro	3

Senegal	3
Andorra	2
United Arab Emirates	2
Albania	2
√Öland Islands	2
Bermuda	2
Cyprus	2
Falkland Islands	2
Gibraltar	2
North Korea	2
Oman	2
Papua New Guinea	2
Paraguay	2
Sierra Leone	2
Suriname	2
South Sudan	2
Chad	2
Samoa	2
Fiji	1
Georgia	1
French Guiana	1
Cayman Islands	1
Madagascar	1
Mozambique	1
Puerto Rico	1
United States Minor Outlying Islands	1
Kosovo	1
Yemen	1

Supplementary Table 3: Structural topic model results from 4301studies (including article titles, abstracts, and keywords) using GBIF-mediated data published from 2003 to 2024. Topics are numbered in descending order by proportion of the entire corpus (i.e., all text analyzed), and including topic name, top 15 words with highest probability in each topic, as well as relative change in topic proportions for the ranges 2016 to 2019 and 2020-2024.

Topic designation	Top 15 words	Topic prevalence all years	Relative change in topics*
Biogeography	gis,gbif,asteracea,dispar,poacea,facil,bioclim,brassicacea,artemisiifolia,cyperacea,ambrosia,republ,poa,micrantha,cgis	0.2%	0.0%
Paleobiology	dispers,diversif,america,biogeograph,region,fossil,reconstruct,climat,clade,time,lineag,south,asia,origin,biogeographi	4.7%	-0.9%
Population structure	popul,genet,divers,structur,glacial,speci,distribut,last,differenti,rang,diverg,pattern,isol,geograph,gene	4.9%	-1.8%
Phylogenetics	speci,phylogenet,morpholog,evolut,studi,genom,genus,group,molecular,hybrid,ecolog,evolutionari,two,differ,diverg	5.8%	-0.9%
Habitat modelling	model,nich,speci,distribut,use,predict,ecolog,environment,variabl,rang,data,differ,approach,method,perform	8.6%	-1.9%
Plamt traits	trait,plant,speci,temperatur,climat,function,increas,size,respons,across,communiti,interact,differ,effect,toler	7.6%	-0.1%
Forest Biology	forest,speci,tree,veget,divers,tropic,rich,plant,region,soil,high,biom,elev,type,dri	4.2%	0.1%
Invasive species	invas,speci,nativ,alien,rang,potenti,spread,risk,plant,invad,introduc,establish,south,introduct,manag	5.1%	-0.7%
Crop science	crop,insect,product,agricultur,cultiv,potenti,control,beetl,use,fruit,palm,america,econom,global	2.9%	0.2%
Conservation management	conserv,area,protect,habitat,biodivers,speci,land,identifi,ecosystem,prioriti,ecolog,use,manag,urban,plan	6.5%	2.2%
Marine	speci,new,record,distribut,sea,marin,island,first,fish,report,ocean,specimen,present,region,collect	7.7%	-1.5%
Species distribution modelling	distribut,suitabl,area,model,habitat,potenti,china,maxent,temperatur,predict,variabl,precipit,factor,environment,studi	7.6%	2.4%
Endemism	endem,mexico,distribut,area,speci,central,california,mexican,state,pine,region,sierra,var,highland,pinus	1.8%	-0.2%
Plant conservation	plant,use,research,wild,resourc,collect,studi,develop,system,review,medicin,provid,situ,inform,tradit	5.2%	-0.8%
Evolution	speci,conserv,distribut,rang,popul,status,extinct,habitat,threaten,assess,list,bird,amphibian,red,mammal	5.1%	0.6%
Biodiversity Informatics	data,speci,biodivers,divers,rich,record,sampl,global,inform,occurr,pattern,databas,use,spatial,map	6.6%	-0.2%
Climate change	climat,chang,speci,futur,distribut,scenario,suitabl,model,current,rang,habitat,will,shift,project,impact	9.2%	2.6%
Regional diversity	europ,mediterranean,european,pollin,speci,plant,peninsula,distribut,northern,pollen,rang,iberian,central,orchid,studi	2.6%	-0.2%

*between 2016-2019 and 2020-2024

Supplementary Table 4: List of topics based on GBIF- assigned science categories

Topic
AGRICULTURE
BIODIVERSITY_SCIENCE
BIOGEOGRAPHY
CITIZEN_SCIENCE
CLIMATE_CHANGE
CONSERVATION
DATA_MANAGEMENT
DATA_PAPER
ECOLOGY
ECOSYSTEM_SERVICES
EVOLUTION
FRESHWATER
HUMAN_HEALTH
INVASIVES
MARINE
PHYLOGENETICS
SPECIES_DISTRIBUTIONS
TAXONOMY
